# Novel, Speedy, and Eco-Friendly Carboxymethyl Cellulose-Nitrogen Doped Carbon Dots Biosensors with DFT Calculations, Molecular Docking, and Experimental Validation

**DOI:** 10.3390/gels10110686

**Published:** 2024-10-24

**Authors:** Hebat-Allah S. Tohamy

**Affiliations:** Cellulose & Paper Department, National Research Centre, 33 El-Bohouth St., Dokki, Giza P.O. Box 12622, Egypt; hs.tohamy@nrc.sci.eg or hebasarhan89@yahoo.com

**Keywords:** microwave carboxymethyl cellulose, biosensor fluorescence, bacterial detection, fungal detection, antibacterial/antifungal activity, nitrogen doped carbon dots

## Abstract

Carboxymethyl cellulose (CMC) was prepared from sugarcane bagasse (SB) in minutes using a novel microwave method. Additionally, nitrogen-doped carbon dots (N–CDs) were synthesized from SB using the same microwave technique. These materials were crosslinked with CaCl_2_ to prepare antibacterial/antifungal hydrogel sensors. In this regard, both CMC@Ca and CMC@Ca-N–CDs exhibited antibacterial activity against *Escherichia coli* (Gram negative), while only CMC@Ca-N–CDs demonstrated antibacterial activity against *Staphylococcus aureus* (Gram positive). Moreover, both materials showed antifungal activity against *Candida albicans*. The molecular docking study demonstrated that CMC@Ca-N–CDs showed good binding with proteins with short bond length 2.59, 2.80, and 1.97 A° for *Escherichia coli*, *Staphylococcus aureus*, and *Candida albicans*, respectively. These binding affinities were corroborated by the observed inhibition zone diameters. Furthermore, fluorescence microscope revealed distinct imaging patterns between Gram-positive and Gram-negative bacteria, as well as pathogenic yeast (fungi). CMC@Ca-N–CDs emitted blue light when exposed to *Escherichia coli* and *Candida albicans* (i.e., CMC@Ca-N–CDs/*Escherichia coli* and *Candida albicans*), whereas it emitted bright-red light when exposed to *Staphylococcus aureus* (i.e., CMC@Ca-N–CDs/*Staphylococcus aureus*). This disparity in the fluorescence-emitted colors is due to the difference in the cell wall of these microorganisms. Additionally, DFT calculations were conducted to substantiate the robust chemical interactions between CMC, Ca^2+^, and N–CDs.

## 1. Introduction

Cellulose, a naturally abundant polymer, can be derived from sustainable sources to mitigate environmental concerns [1,2,3]. Sugarcane bagasse (SB), an agricultural waste product, represents a promising feedstock for cellulose extraction [4,5]. By transforming this abundant and often underutilized resource, we can address the pressing issues of SB accumulation and the harmful practice of open burning. Extracting cellulose from SB not only provides a renewable and eco-friendly source of this valuable material but also contributes to a cleaner environment by diverting SB from landfills and reducing greenhouse gas emissions [6]. Cellulose holds immense potential for diverse applications across scientific fields. However, its inherent recalcitrance often hinders its direct conversion into valuable products. To overcome this challenge, researchers have focused on modifying cellulose’s structure to enhance its processability and expand its utility [6]. Carboxymethylation is a key strategy in this regard [7,8]. By introducing carboxymethyl groups into the cellulose backbone, its solubility and compatibility with other materials are significantly improved, resulting in the formation of carboxymethyl cellulose (CMC) [9,10]. This derivative offers a sustainable alternative to synthetic polymers, broadening the application spectrum of cellulose-based materials. Conventional methods to enhance cellulose’s properties, such as carboxymethylation, often involve lengthy reaction times, limiting their industrial feasibility [11,12]. Our novel microwave-assisted synthesis has emerged as a promising technique to accelerate the carboxymethylation process, drastically reducing reaction times from hours to mere minutes.

Carboxymethyl cellulose (CMC), derived from sustainably sourced SB, is a key ingredient in the fabrication of hydrogels [13,14]. Hydrogels, three-dimensional polymeric networks capable of absorbing and retaining significant amounts of water, have garnered considerable attention due to their versatile applications in various fields, including biomedical, agriculture, and environmental sectors [10,15]. Cellulose-based hydrogels, in particular, offer several advantages such as biocompatibility, biodegradability, and abundance [4]. The preparation of hydrogels using CMC enhances gel formation properties. Moreover, the hydrophilic nature of CMC contributes to the hydrogel’s ability to create a moist environment, which is crucial for many applications [4,9]. One of the most promising areas of hydrogel research is the development of antibacterial materials. Cellulose-based hydrogels, including those containing CMC, have exhibited intrinsic antimicrobial properties [16,17,18]. These properties can be further enhanced by incorporating antimicrobial agents. Such antibacterial hydrogels hold great potential for packaging applications.

Carbon dots (CDs), renowned for their exceptional optical properties, are emerging as promising candidates for enhancing hydrogel performance [19,20]. By incorporating CDs into cellulose-based hydrogels, researchers can imbue these materials with antibacterial capabilities and biosensing functions. The interesting part is that, depending on the bacterium they are linked to, their luminescence can change color [21]. The unique optical characteristics of CDs, including their tunable emission spectra and high quantum yield, enable them to act as efficient antibacterial agents. Upon exposure to bacteria, CDs can generate reactive oxygen species (ROS) that disrupt microbial cell membranes, leading to cell death [9,22,23,24,25,26]. Furthermore, the embedded N–CDs can serve as fluorescent probes for monitoring of bacteria [9,27,28]. Determining the identity of bacteria accurately is essential for both diagnosing diseases and creating potent medications. Interestingly, tiny fluorescent CDs are sensitive to the type of bacteria they come into contact with, changing both color and brightness. Because of this, they are an effective tool for separating various bacteria, even in combinations. Numerous microorganisms, including *Bacillus subtilis*, *E. coli*, *S. aureus*, and yeast, have already been successfully imaged by researchers using CDs [13,29,30]. In a previous study, gelatin CDs, created through hydrothermal synthesis, bind to various cells including *E. coli*, *S. aureus*, and *Candida* species. These CDs emit different colors when exposed to light, based on the light’s wavelength [31].

Food contaminated with antibiotic-resistant bacteria (such as *Escherichia coli* and *Staphylococcus aureus*) poses a significant public health risk as these bacteria can transfer their resistance to other pathogens, potentially rendering treatments for severe bacterial infections ineffective. The problem of antibiotic resistance among foodborne pathogens has become increasingly prevalent over the past decade [32]. At the same time, *Candida albicans* is a single-celled fungus that naturally inhabits human skin, digestive system, and vagina, though it can become pathogenic under certain conditions [33,34,35]. Under specific conditions, the fungus *Candida albicans*, normally harmless when in balance, can experience excessive growth within the human body, leading to a fungal infection known as candidiasis [36,37]. Thus, detecting and combating *Escherichia coli*, *Staphylococcus aureus*, and *Candida albicans* is crucial for human safety [38,39]. Wang et al. found that nitrogen-rich CDs (N–CDs) are exceptionally effective catalysts for the electrochemical reduction of oxygen [40]. The increased nitrogen content of the N–CDs is associated with red light emission [41,42]. These N–CDs’ red light may penetrate far into tissues without being obscured by the body’s inherent glow [43,44,45]. In addition, CDs have recently emerged as a promising tool for fluorescent imaging of *Candida albicans* cells [46]. Because of this, we will use greener alternative N–CDs manufactured from recycled SB with red emissive properties, instead of relying on conventional reducing agents. By employing an environmentally friendly microwave method, we can prepare N–CDs from SB agricultural waste [19,47].

The combination of cellulose hydrogels and N–CDs creates a synergistic effect, where the hydrogel provides a biocompatible and hydrophilic environment for N–CDs dispersion, while the N–CDs impart antibacterial and sensing properties [6,9]. This integration can lead to the development of intelligent hydrogels capable of not only preventing bacterial infections but also providing early warning signals of microbial contamination [9,31]. By embedding N–CDs within the cellulosic hydrogel matrix, it becomes possible to create intelligent packaging materials capable of detecting various foodborne contaminants and spoilage indicators. By leveraging the sustainable production of CMC from SB, researchers can develop a new generation of cellulose-based hydrogels with improved properties and expanded applications. This approach aligns with the growing demand for environmentally friendly and high-performance materials.

## 2. Results and Discussion

**Figure 1:**
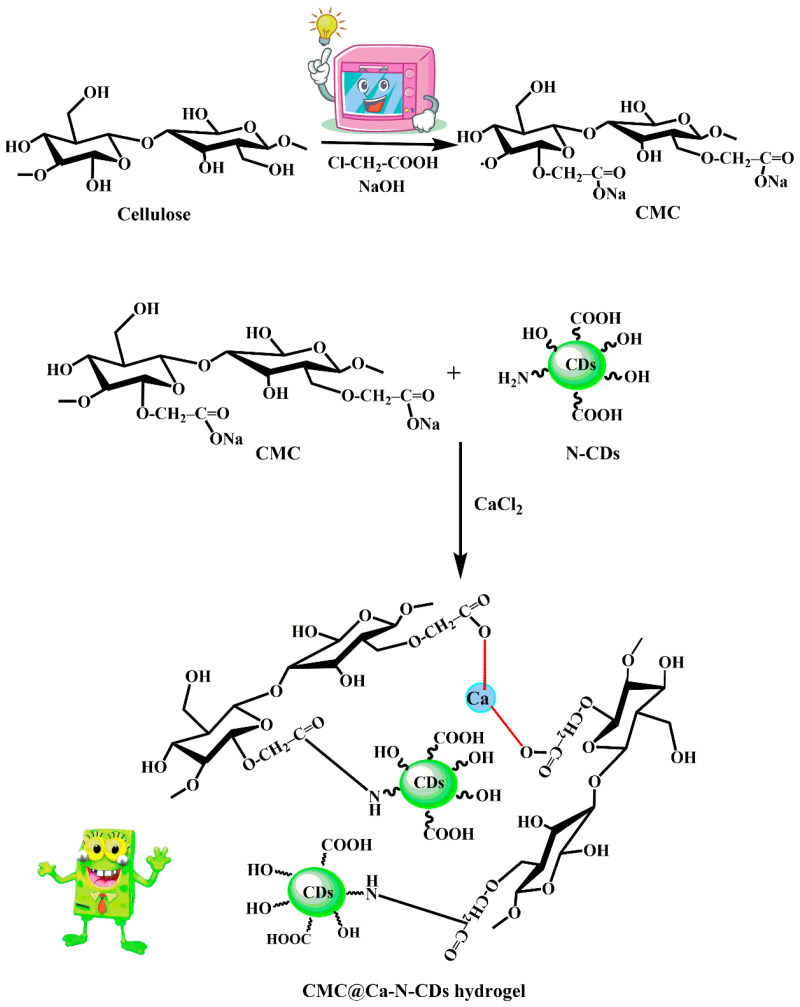
Reaction mechanism for synthesizing CMC@Ca-N–CDs hydrogel.

Figure 1 outlines the proposed mechanism for the grafting and chemical crosslinking reactions. The CMC contains COO-Na^+^ groups, while N–CDs possess NH_2_ groups. These functional groups can undergo condensation reaction, forming an amide bond (-CO–NH-) and releasing a water molecule as a byproduct. Additionally, Ca^2+^ are attracted to the COO^–^ on the CMC chains, leading to the formation of ionic bonds and the creation of crosslinks between the CMC polymer chains [48].

### 2.1. Fourier Transform Infrared Spectroscopy (FTIR) Spectra

The surface chemistry of the CMC@Ca and CMC@Ca-N–CDs hydrogels was generally investigated using FTIR. The cellulose and CMC exhibited absorption bands between 3221–3344 cm^−^^1^ (O–H), 1450–1455 cm^−^^1^ (C–O bending), 1315–1386 cm^−^^1^ (C–H bending), 1029–1037 cm^−^^1^ (C–O–C of pyranose ring vibration), and 882–896 cm^−^^1^ (ꞵ-glycosidic linkage) [11,12]. The peak at 1635 cm^−^^1^ observed in cellulose is attributed to the OH bending of adsorbed water. In contrast, the new peaks observed in CMC at 1745 cm^−^^1^ are associated with the C=O stretching vibration and the anti-symmetric and symmetric stretching vibrations of the COO– group in CMC [8,12]. The N–CDs exhibited absorption peaks at 3432 cm^−^^1^ (N–H), 3330 cm^−^^1^ (O–H), 1677 cm^−^^1^ (C=O and Amide I), 1594 cm^−^^1^ (Amide II), 1459 cm^−^^1^ (C=C), 1303 cm^−^^1^ (O=C–O), 1212 cm^−^^1^ (C–O–C), and 1149 cm^−^^1^ (C–N) groups [4,19]. The CMC@Ca showed a broad peak at 3346 cm^−^^1^ attributed to O–H, while the CMC@Ca-N–CDs exhibited a broad peak at 3319 cm^−^^1^, attributed to N–H/O–H [20]. The sharp peak at ∼2225 cm^−^^1^, typically associated with atmospheric CO_2_ in N–CDs, appeared at 2244 cm^−^^1^ in CMC@Ca-N–CDs. Moreover, the new peak at 2169 cm^−^^1^ indicated the stretching of the NCO group, which was not observed in N–CDs/CMC, suggesting that the modification led to the formation of this NCO group [29,49].

**Figure 2:**
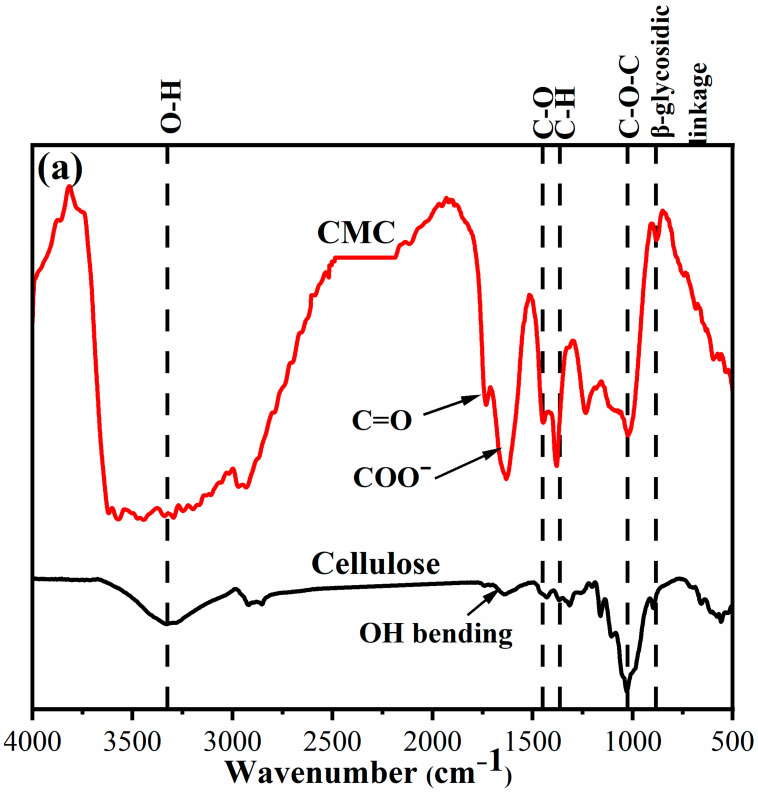

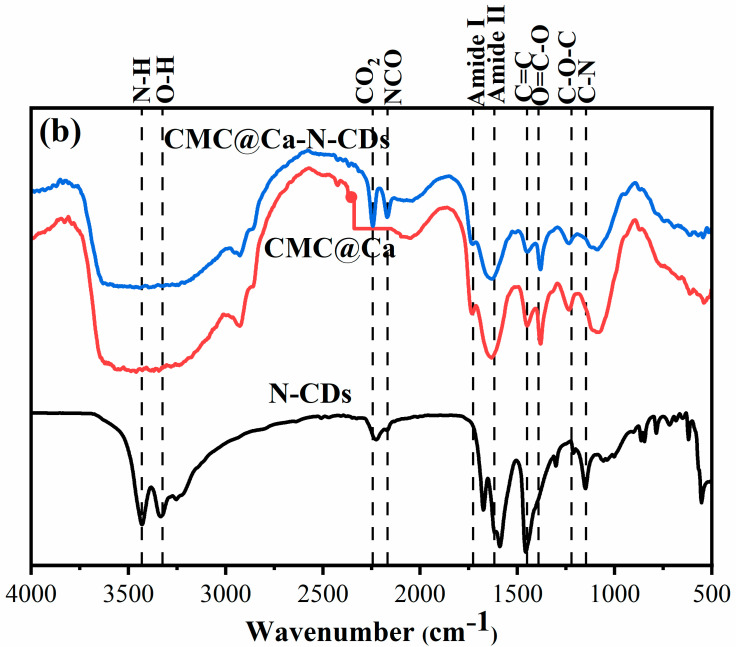
FTIR spectra of (**a**) cellulose, and CMC and (**b**) N–CDs, CMC@Ca, and CMC@Ca-N–CDs.

For CMC@Ca, there were no significant differences observed in the FT-IR spectra of CMC and for CMC@Ca hydrogel. No new peaks were observed in the CMC@Ca hydrogel. This could be attributed to the fact that the crosslinking process involved only the substitution of Na^+^ in CMC with Ca^2+^ from CaCl_2_. The ionic crosslinking of Ca^2+^ with the CMC chain resulted in a slight shift in the wavenumber due to the bidentate coordination of the carboxylate group to Ca^2+^ [50]. The MHBSs were calculated to be 0.76 and 1.56 for CMC@Ca and CMC@Ca-N–CDs, respectively, indicating stronger H-bonding between CMC@Ca-N–CDs compared to CMC@Ca. This observation is further supported by the shift of the O–H stretching peak from 3334 cm^−^^1^ in CMC@Ca to 3319 cm^−^^1^ in CMC@Ca-N–CDs (Figure 2), suggesting stronger intermolecular H-bonding [11].

### 2.2. DFT Calculations

**Figure 3:**
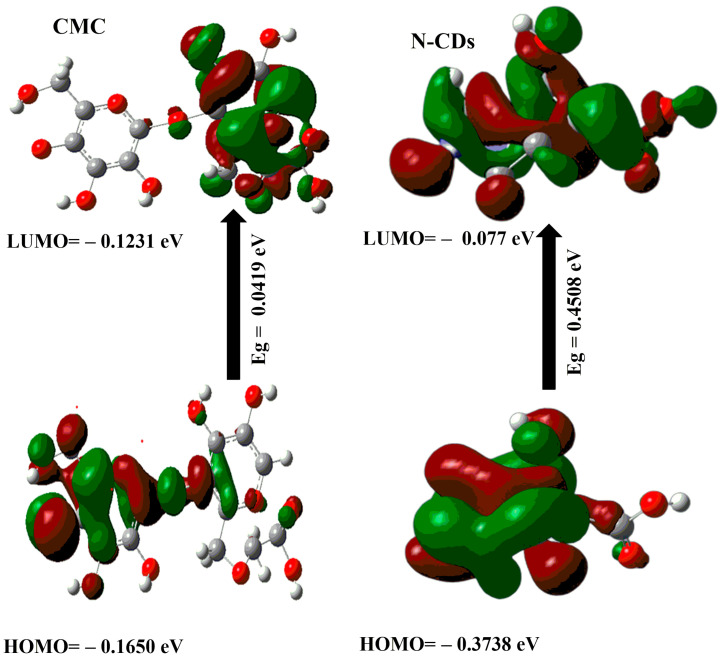

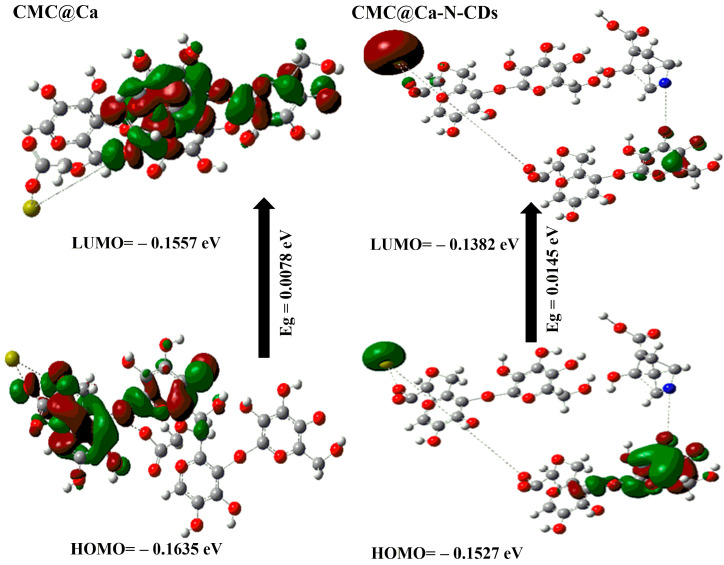
The gap energies (HOMO–LUMO) (eV) were calculated for the CMC, N–CDs, CMC@Ca, and CMC@Ca-N–CDs using DFT B3LYP/6–31G (d).

DFT calculations were employed to study the stability of the CMC, N–CDs, CMC@Ca, and CMC@Ca-N–CDs. From Figure 3 and Table 1, the following results were obtained:

(a) The μ for CMC@Ca-N–CDs (i.e., 77.763 Debye) is higher than that of CMC@Ca (i.e., 15.304 Debye). This can be attributed to the presence of more O and N atoms from N–CDs, which increase the electronegativity of CMC@Ca-N–CDs compared to CMC@Ca [51].
(b) The calculated E_g_ for CMC@Ca and CMC@Ca-N–CDs is the lowest (i.e., 0.0078 and 0.0145 eV) compared to CMC and N–CDs (i.e., 0.0419 and 0.4508 eV). This indicates a strong chemical interaction between CMC, Ca^2+^, and N–CDs [1,52]. The positive charge on the nitrogen atom of N–CDs can play a role in the interactions between N–CDs and other molecules, such as the repulsive interaction with Ca^2+^.
  The lower E_g_ observed in CMC@Ca compared to CMC@Ca-N–CDs can be attributed to the absence of N–CDs, which in turn means the absence of repulsion with Ca^2+^. Without these N–CDs, the E_g_ in CMC@Ca may be narrower (i.e., no repulsion). However, it is important to note that the E_g_ of CMC@Ca-N–CDs is still lower than CMC and N–CDs. This is a good proof of the CMC@Ca-N–CD reactivity and enhanced fluorescent properties compared to N–CDs.
(c) The E_T_ of CMC@Ca-N–CDs (1.998 au) is higher than that of CMC@Ca (−3542.25 au). This can be attributed to the formation of new amide bonds between N–CDs and CMC within the CMC@Ca-N–CDs hydrogel, which introduces additional bond energies.
(d) The CMC@Ca is much softer (128.205 eV) than the CMC@Ca-N–CDs (68.965 eV). This suggests that CMC@Ca likely forms a more open and flexible network due to the nature of CMC and the ionic interactions with Ca^2+^. The interactions between CMC chains and Ca^2+^ might contribute to a more elastic structure. In contrast, CMC@Ca-N–CDs contain N–CDs, which form amide bonds, leading to a stiffer hydrogel.
(e) The calculated ΔN_max_ for CMC@Ca-N–CDs (−20.062) is higher than that of CMC@Ca (−40.923). This can be attributed to the presence of N–CDs, which introduce more electronegative atoms in the CMC@Ca-N–CDs structure.

### 2.3. Molecular Docking Study

**Figure 4:**
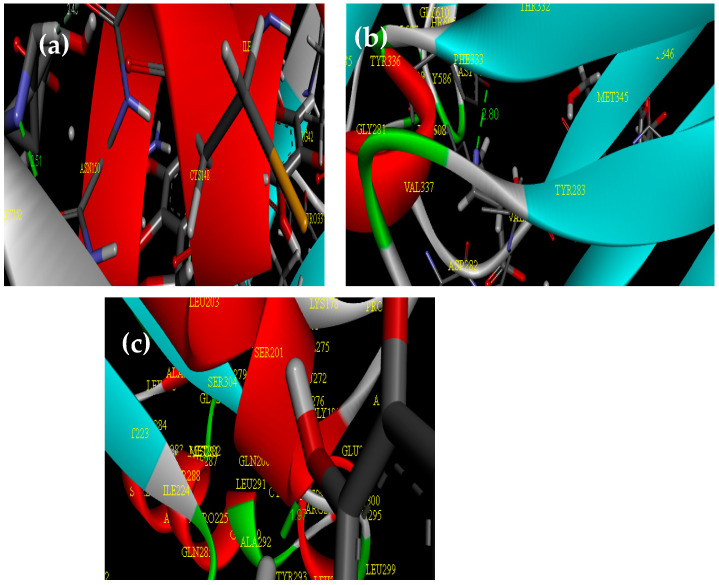
The biological activity of CMC@Ca-N–CDs as a ligand against (**a**) *Escherichia coli* PDB (3ZH7), (**b**) *Staphylococcus aureus* PDB (7BGD), and (**c**) pathogenic yeast (fungi): *Candida albicans* (8GQ3).

Figure 4 illustrates the biological activity of CMC@Ca-N–CDs as a ligand against *Escherichia coli* PDB (3ZH7), *Staphylococcus aureus* PDB (7BGD), and pathogenic yeast (fungi): *Candida albicans* (8GQ3) as a receptor. The CMC@Ca-N–CDs exhibited a strong binding affinity with proteins, characterized by short bond lengths of 2.59, 2.80, and 1.97 A° for *Escherichia coli* (Figure 4a), *Staphylococcus aureus* (Figure 4b), and *Candida albicans* (Figure 4c), respectively. These binding affinities were corroborated by the observed inhibition zone diameters. The study’s results support previous research demonstrating the material’s antimicrobial properties. By forming shorter, stronger bonds with proteins, the material’s reactive ligand effectively disrupts microbial function, ultimately leading to the microbe’s demise [52].

### 2.4. Antibacterial and Antifungal Activity

**Figure 5:**
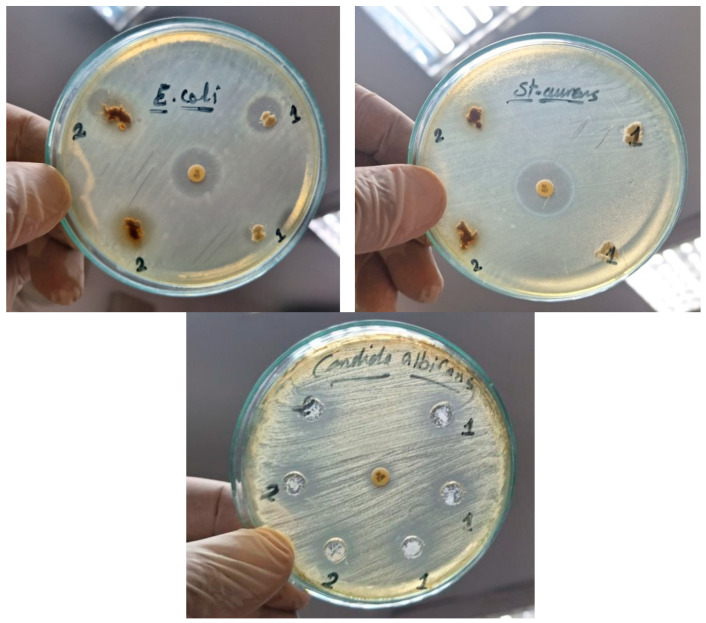
Antibacterial/antifungal activity of the CMC@Ca (denoted as 1) and CMC@Ca-N–CDs (denoted as 2) against *Escherichia coli*, *Staphylococcus aureus*, and *Candida albicans*.

The antibacterial activity of CMC and N–CDs can be attributed to their unique properties. N–CDs, in particular, are known to generate ROS and interact directly with bacterial cell components, leading to cell death [22,23,24,26]. While CMC may exhibit some inherent antimicrobial properties, its activity is likely less pronounced compared to N–CDs [16,17,18]. The interaction between N–CDs and CMC through functional group interactions can contribute to the formation of a composite hydrogel with enhanced properties. However, this interaction does not necessarily diminish the antibacterial activity of N–CDs. The N–CDs can still retain their ability to generate ROS and interact with bacterial cells, even when incorporated into the hydrogel. The findings revealed that CMC@Ca (denoted as 1) and CMC@Ca-N–CDs (denoted as 2) exhibited antibacterial activity against Gram-negative bacteria such as *Escherichia coli* (with inhibition zones 11 mm for CMC@Ca to 15 mm for CMC@Ca-N–CDs). In addition, only CMC@Ca-N–CDs exhibited antibacterial activity against Gram-positive bacteria such as *Staphylococcus aureus* (with no inhibition zone for CMC@Ca and inhibition zone 13 mm for CMC@Ca-N–CDs). The inhibition zones against *Candida albicans* were 15 mm for CMC@Ca and 17 mm for CMC@Ca-N–CDs (Figure 5, Table 2). The observed antibacterial properties of N–CDs are likely attributed to their oxygen and nitrogen groups, which interact with bacterial components like lipids, proteins, and DNA/RNA through hydrogen bonding, π-π interactions, and electrostatic forces. Additionally, N–CDs appear to regulate the production of reactive oxygen species, a mechanism potentially inherited from bacterial surface proteins [53]. The CMC@Ca-N–CDs hydrogel exhibited the strongest antimicrobial activity against *Candida albicans*, followed by *Escherichia coli* and *Staphylococcus aureus*. These findings highlight the CMC@Ca-N–CDs’ potential as a promising antibacterial/antifungal agent for combating a wide range of pathogens.

### 2.5. CMC@Ca-N–CD-Based Fluorescent Hydrogel as a Probe for Imaging of Bacteria and Fungi

**Figure 6:**
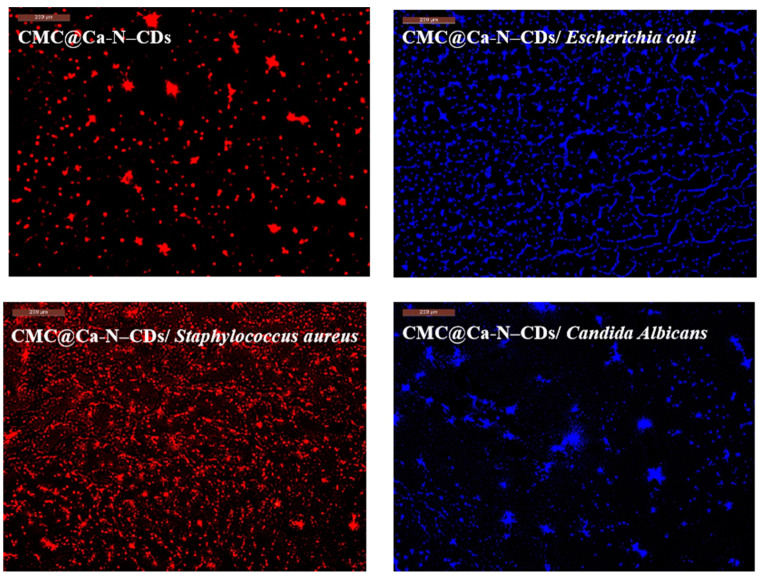
Fluorescence microscope for CMC@Ca-N–CDs before bacteria, CMC@Ca-N–CDs/*Escherichia coli*, CMC@Ca-N–CDs/*Staphylococcus aureus*, and CMC@Ca-N–CDs/*Candida albicans*.

Given the critical threat bacteria pose to both the environment and human health, the development of sensitive detection methods is imperative [1,54,55]. Our previously synthesized N–CDs fulfill the necessary criteria for a robust fluorescent probe [4,15]. Consequently, we investigated the potential of these materials for identifying bacteria. The fluorescence of CMC@Ca-N–CDs before bacteria, CMC@Ca-N–CDs/*Escherichia coli*, CMC@Ca-N–CDs/*Staphylococcus aureus*, and CMC@Ca-N–CDs/*Candida albicans* was observed using a fluorescence microscope and the intense red fluorescence was observed in CMC@Ca-N–CDs before bacterial exposure (Figure 6). The prepared CDs are readily absorbed into cells due to their hydrophilic nature and the presence of reactive functional groups, including carbonyl, hydroxyl, and carboxylic acid [13,56]. After bacterial cells harvesting, the CMC@Ca-N–CDs emitted blue light in CMC@Ca-N–CDs/*Escherichia coli*, while it was bright-red light in CMC@Ca-N–CDs/*Staphylococcus aureus* (Figure 6). Despite being cultured in the same neutral environment, Gram-positive (i.e., *Staphylococcus aureus*) and Gram-negative bacteria (i.e., *Escherichia coli*) exhibit distinct cell wall structures. *Staphylococcus aureus* possess teichoic acid within their cell walls; this component is absent in the cell walls of *Escherichia coli* [32]. This structural difference, combined with the presence of hydrogen ions in acidic conditions, can influence the interaction between the CMC@Ca-N–CDs hydrogel and the bacterial cells. The *Staphylococcus aureus* possess more negatively charged teichoic acid on their surface compared to the lipopolysaccharides found in *Escherichia coli*. This difference creates a more favorable environment for the electrostatic attraction and binding of positively charged molecules to the surface of *Staphylococcus aureus*, resulting in the observed smaller and more intense red fluorescent spots compared to CMC@Ca-N–CDs hydrogel before bacterial interaction [30,57]. The observed change in fluorescence color from red to blue in the CMC@Ca-N–CDs/*Escherichia coli* can be attributed to the distinct cell wall structure of this bacterium [58,59,60,61,62,63]. The lipopolysaccharide-rich outer membrane of *Escherichia coli* may hinder the direct interaction between N–CDs and the *Escherichia coli* cell wall, resulting in a different fluorescence response. This could be due to factors such as the shielding effect of the outer membrane, which may prevent N–CDs from penetrating the cell wall and interacting with intracellular components. Alternatively, the lipopolysaccharide molecules themselves may interfere with the fluorescence properties of N–CDs, leading to a change in the emitted light color [58].

The CMC@Ca-N–CDs, characterized by their hydrophilic nature due to the presence of oxygen and nitrogen atoms, are efficiently internalized into *Candida albicans* cells via endocytosis. This is evidenced by the blue fluorescence emitted by the treated cells, indicating successful cellular uptake. The interaction of CMC@Ca-N–CDs with the cell membrane facilitates the analysis of *Candida albicans* metabolism [64].

### 2.6. Morphological Observations

**Figure 7:**
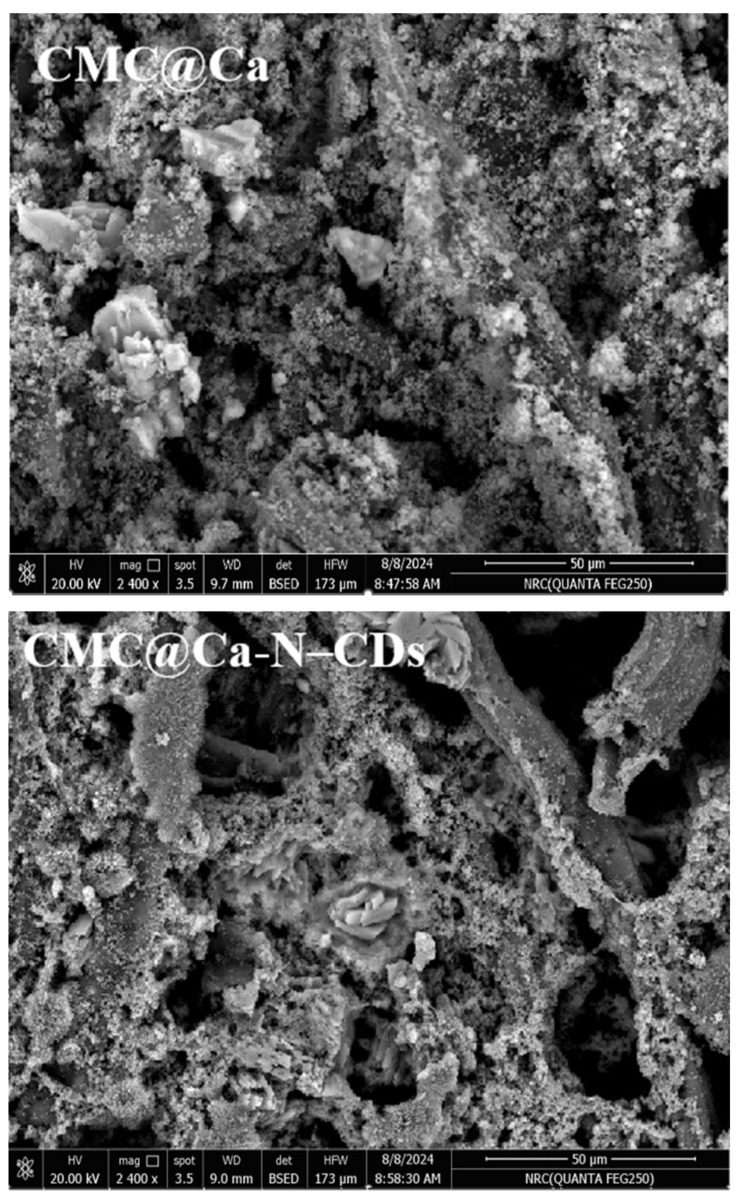
SEM analysis of CMC@Ca and CMC@Ca-N–CDs hydrogels.

Figure 7 depicts the surface morphology of the CMC@Ca and CMC@Ca-N–CDs hydrogels. It was observed that the pore size of CMC@Ca hydrogel, which ranged between 1.87 and 3.57 µm, decreased for the CMC@Ca-N–CDs hydrogel to a range of 1.21–1.83 µm, indicating stronger chemical bonds between CMC, Ca^2+^, and N–CDs.

## 3. Conclusions

The domestic microwave method was used to prepare CMC and N–CDs from SB using an eco-friendly, fast, and low-cost simple technique. The prepared N–CDs were then utilized to create the CMC@Ca-N–CDs hydrogel. The presence of N–CDs enhanced the antibacterial activity of CMC@Ca-N–CDs against both Gram negative and positive bacterial, as well as fungi, compared to CMC@Ca. The study demonstrated that the interaction between CMC@Ca-N–CDs and bacteria is influenced by cell wall structure. Gram-positive bacteria, rich in negatively charged teichoic acid, strongly bound to the cationic groups of the N–CDs, leading to rapid penetration of the cell wall and subsequent red fluorescence. In contrast, fungi emitted blue color when reacted with CMC@Ca-N–CDs. Computational modeling (DFT calculations and molecular docking) confirmed a robust binding interaction between the bacteria or fungi and the CMC@Ca-N–CDs hydrogel.

## 4. Materials and Methods

### 4.1. Materials

Bagasse agrowastes (SB) was received from the Quena Company for Paper Industry in Egypt. The monochloro-acetic acid, urea, and calcium chloride were purchased from Sigma-Aldrich (St. Louis, MO, USA). All materials were used as received without further purification.

### 4.2. Preparation of Cellulose

SB (150 g) was initially prehydrolyzed using dilute hydrochloric acid in an autoclave at 120 °C for 2 h. Subsequently, the prehydrolyzed material was subjected to alkaline treatment with sodium hydroxide (20 g of NaOH in 300 mL water) at 170 °C for 2 h, resulting in a brown, cotton-like pulp. To eliminate lignin, the pulp (80 g) was bleached with HClO_2_ (3%; 2.4 g in 4750 mL water) in an acetic acid medium at 80 °C for 2 h, maintaining a pH of 1–3 through acetic acid addition to obtain pure α-cellulose [11].

### 4.3. Preparation of Carboxymethyl Cellulose in Microwave

The prepared cellulose (15 g) was mixed with 30% NaOH solution and monochloro acetic acid (18 g), followed by heating in the microwave until complete dissolution. The prepared CMC was precipitated with 70% methanol, filtered, and dried at oven.

### 4.4. Preparation of Nitrogen Carbon Dots

A mixture containing 30 mg of SB, 70 mg of NaOH, and 2400 mg of urea dissolved in 100 mL of water was homogenized for 30 min before being frozen overnight. The thawed solution was then subjected to ultrasonic treatment for 2 min, followed by microwave heating at 700 W for approximately 7 min [4,5].

### 4.5. Preparation of CMC@Ca-N–CDs

CMC hydrogels were prepared by dissolving 4 g CMC in 50 mL H_2_O containing 15% CaCl_2_ and 10% N–CDs and keep the mixture stand for 24 h at 40 °C, resulting in CMC@Ca-N–CDs hydrogel. A control hydrogel, CMC@Ca, was produced similarly but without N–CDs [50].

### 4.6. Carbon Dots for Bacterial and Fungal Sensing

Bacterial cells were harvested during their most active growth period (exponential phase) for subsequent examination under a confocal microscope. The cells-CMC@Ca-N–CDs-conjugates were washed with double distilled water and their images were measured by using a fluorescence microscope (Jasco Intern. Co., Tokyo, Japan). The CMC@Ca-N–CDs showed different imaging between Gram-positive and Gram-negative bacteria and pathogenic yeast (fungi).

### 4.7. Characterization and Analysis

#### 4.7.1. SEM

SEM images were captured using a Quanta/250-FEG microscope (Elecmi, Zaragoza, Spain) at 30 kV acceleration voltage.

#### 4.7.2. Fluorescence Microscope

Fluorescence measurements were conducted using a Jasco FP-6500 spectrofluorometer (Jasco Intern. Co., Tokyo, Japan) equipped with a 150-watt xenon lamp.

#### 4.7.3. Fourier-Transform Infrared (FTIR) Spectra

FTIR spectra were obtained using a Mattson 5000 spectrometer (Unicam, Somerset, UK) and KBr pellet method. The mean hydrogen bond strength was determined through Equation (1).

(1) $$\mathrm{MHBS}=\frac{A_{OH}}{A_{CH}}$$

where A_OH_ and A_CH_ refer to the FTIR absorbance of the OH and CH peaks, respectively [10,11].

#### 4.7.4. DFT Calculations

Density functional theory (DFT) calculations were performed using Gaussian 09 W, B3LYP functional, and 6–31 G(d) basis set exhausted by the Berny technique. Different parameters were investigated via DFT calculations, including some of the optimized geometries and ground state energies, including total energy (*E_T_*), the energy of the highest occupied MO (*E_HOMO_*), the energy of the lowest unoccupied MO (*E_LUMO_*), the energy gap (*E_g_*), the dipole moment (μ), the absolute electronegativity (χ), the chemical potential (Pi), the absolute hardness (η), the absolute softness (σ), the chemical softness (S), and the additional electronic charge (Δ*N_max_*) [65,66,67,68,69,70,71,72,73,74].

(2) $${\;E}_{gap}=(E_{LUMO}-E_{HOMO})$$

(3) $$\chi =\frac{–(E_{HOMO}+E_{LUMO})}{2}$$

(4) Pi=−χ

(5) $$\eta =\frac{\left(E_{LUMO}+E_{HOMO}\right)}{2}$$

(6) $$\sigma =\frac{1}{\eta }$$

(7) $$S=\frac{1}{2\eta }$$

(8) $$\Delta N_{max}=\frac{-Pi}{\eta }$$

#### 4.7.5. Molecular Docking

The molecular docking of CMC@Ca-N–CDs against *Escherichia coli* PDB (3ZH7), *Staphylococcus aureus* PDB (7BGD), and *pathogenic yeast* (fungi): *Candida albicans* (8GQ3) as a receptor. The protein complex was fabricated using standard bond length, with the Gaussian 09 W and detected by discovery Studio Client (version 4.2).

## Figures and Tables

**Table 1:** The quantum chemical parameters of CMC, N–CDs, CMC@Ca, and CMC@Ca-N–CDs.

DFT B3LYP/6–31G (d)	CMC	N–CDs	CMC@Ca	CMC@Ca-N–CDs
*E_LUMO_* (eV)	−0.1231	0.077	−0.1557	−0.1382
*E_HOMO_* (eV)	−0.1650	−0.3738	−0.1635	−0.1527
*E_g_* (eV)	0.0419	0.4508	0.0078	0.0145
*E_T_* (au)	−1134.555	−244.29	−3542.25	1.998
χ (eV)	0.144	0.148	0.1596	0.1454
μ (Debye)	11.14	2.98	15.304	77.763
η (eV)	0.0209	0.2254	0.0039	0.00725
σ (eV)	47.732	4.436	256.41	137.931
S (eV)	23.866	2.218	128.205	68.965
Δ*N_max_*	−6.675	−0.6583	−40.923	−20.062

**Table 2:** The diameter of the inhibition zone from each microbe with CMC@Ca and CMC@Ca-N–CDs along with their average values.

Microbe	CMC@Ca	Average	CMC@Ca-N–CDs	Average
*Escherichia coli*	10, 12	11	15, 15	15
*Staphylococcus aureus*	–	–	12, 14	13
*Candida albicans*	14, 16	15	16, 18	17

## Data Availability

The original contributions presented in the study are included in the article material; further inquiries can be directed to the author.
